# *RUNX2* Mutation Impairs 1α,25-Dihydroxyvitamin D_3_ mediated Osteoclastogenesis in Dental Follicle Cells

**DOI:** 10.1038/srep24225

**Published:** 2016-04-12

**Authors:** X. Z. Wang, X. Y. Sun, C. Y. Zhang, X. Yang, W. J. Yan, L. H. Ge, S. G. Zheng

**Affiliations:** 1 Department of Preventive Dentistry, Peking University School and Hospital of Stomatology, 22 Zhongguancun Avenue South, Haidian District, Beijing 100081, PR China; 2 Department of Stomatology, Dongzhimen Hospital Beijing University of Chinese Medicine, 5 Haiyuncang Alley, Dongcheng District, Beijing 100700, PR China; 3 Outpatient Center, Peking University School and Hospital of Stomatology, 37A Xishiku Street, Xicheng District, Beijing 100034, PR China; 4 Department of Pediatric Dentistry, Peking University School and Hospital of Stomatology, 22 Zhongguancun Avenue South, Haidian District, Beijing 100081, PR China

## Abstract

Cleidocranial dysplasia (CCD), a skeletal disorder characterized by delayed permanent tooth eruption and other dental abnormalities, is caused by heterozygous *RUNX2* mutations. As an osteoblast-specific transcription factor, *RUNX2* plays a role in bone remodeling, tooth formation and tooth eruption. To investigate the crosstalk between *RUNX2* and 1α,25-dihydroxyvitamin D3 (1α,25-(OH)_2_D_3_) in human dental follicle cells (hDFCs) during osteoclast formation, we established a co-culture system of hDFCs from CCD patient and healthy donors with peripheral blood mononuclear cells (PBMCs). Expression of the osteoclast-associated genes and the number of TRAP^+^ cells were reduced in CCD hDFCs, indicating its suppressed osteoclast-inductive ability, which was reflected by the downregulated RANKL/OPG ratio. In addition, 1α,25-(OH)_2_D_3_-stimulation elevated the expression of osteoclast-related genes, as well as *RANKL* mRNA levels and RANKL/OPG ratios in control hDFCs. Conversely, *RUNX2* mutation abolished this 1α,25-(OH)_2_D_3_-induced *RANKL* gene activation and osteoclast formation in CCD hDFCs. Therefore, *RUNX2* haploinsufficiency impairs dental follicle-induced osteoclast formation capacity through RANKL/OPG signaling, which may be partially responsible for delayed permanent tooth eruption in CCD patients. Furthermore, this abnormality was not rescued by 1α,25-(OH)_2_D_3_ application because 1α,25-(OH)_2_D_3_-induced RANKL activation in hDFCs is mediated principally via the *RUNX2*-dependent pathway.

Cleidocranial dysplasia (CCD; MIM 119600), a rare hereditary autosomal dominant skeletal disorder, has been demonstrated to be caused by heterozygous mutations in *RUNX2* gene (also known as *CBFA1*, *PEBP2A1*, *OSF2*, and *AML3*)^1^. The clinical manifestations of CCD are highly variable, characterized by hypoplasia or absence of the clavicle, frontal bossing, brachycephaly, delayed or absent closure of the fontanelle and cranial sutures, multiple supernumerary teeth, prolonged retention of the deciduous dentition, delayed eruption of permanent teeth and other abnormalities of skeletal and dental development^2^,^3^.

As an osteoblast-specific transcription factor, *RUNX2*, which maps to human chromosome 6p21^3^, regulates the expression of all major osteoblast-related genes, such as osteocalcin, BSP, type I collagen and osteopontin^4^,^5^. It is not only involved in bone and cartilage development and maintenance, but is also essential for osteoblast differentiation, chondrocyte maturation and osteoclastogenesis^6^,^7^. *RUNX2* is also expressed in dental epithelium, dental papilla and dental follicles during tooth development and eruption^8^,^9^.

Delayed eruption of permanent teeth is the most frequently noticed phenomenon in CCD. Heterozygous *RUNX2* mutant mice exhibit significantly delayed tooth eruption caused by a time-specific lack of osteoclasts on the bone surface facing the developing tooth^10^. It is widely accepted that tooth eruption is orchestrated by dental follicle (DF) with precise regulation of the osteoclast–osteoblast interaction^11^,^12^. Creation of the eruption pathway requires recruitment of sufficient osteoclasts leading to alveolar bone resorption in a process that is predominantly mediated by spatiotemporal-specific expression of colony stimulating factor 1 (CSF-1), ligand RANK ligand (RANKL), and osteoprotegerin (OPG)^11^,^12^. Furthermore, evidence suggests that bone remodeling is influenced by *RUNX2* signaling via the RANKL/OPG and/or RANK/RANKL systems, a mechanism that may also participate in the control of tooth eruption^5^,^15^.

1α,25-dihydroxyvitamin D_3_ (1α,25-(OH)_2_D_3_) functions by binding to the nuclear vitamin D receptor (VDR) to maintain calcium homeostasis and bone metabolism^16^,^17^. The effects of 1α,25-(OH)_2_D_3_ in the bone and root resorption processes are based on its direct induction of osteoblast-mediated osteoclastogenesis^18^,^19^.

Human dental follicle cells (hDFCs) are multipotent and thought to provide a similar function as provided by osteoblasts in bone^20^,^21^. Functional analysis of the crosstalk between *RUNX2* and 1α,25-(OH)_2_D_3_ in hDFCs would further reveal the mechanism underlying the dental abnormalities in CCD patients.

In this study, we first aimed to characterize hDFCs from a CCD patient (a natural human model of *RUNX2* gene mutation) by comparing their morphology and proliferative capacity with hDFCs from unaffected control individuals. We then investigated the expression of factors associated with osteoclast differentiation by co-culturing hDFCs and peripheral blood mononuclear cells (PBMCs) to confirm that the reduced osteoclast-inductive capacity of hDFCs resulted from *RUNX2* mutation.

## Results

### Clinical Manifestation of the CCD Patient

**Figure 1:**
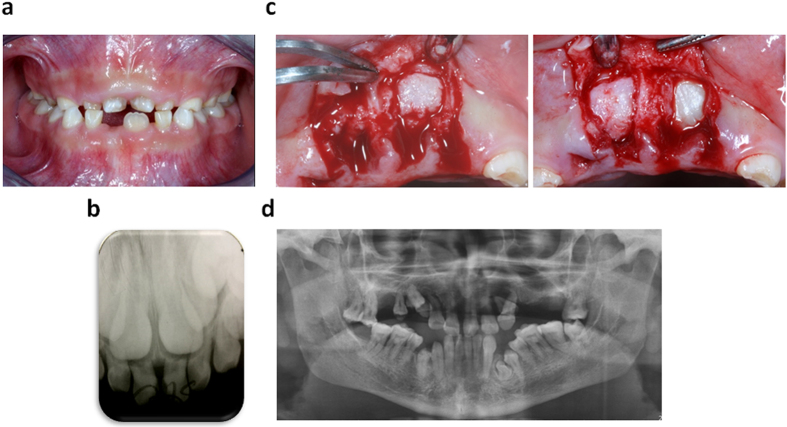
Retained deciduous teeth and multiple impacted permanent teeth in cleidocranial displasia patient. (**a**) Intraoral photograph pre-treatment. (**b**) Periapical film of retained primary maxillary incisors and their successors. (**c**) Unerupted maxillary central incisors covered by dental follicle were surgically exposed. (**d**) Panoramic radiograph of the patient’s father.

The patient in this study manifested a typical CCD appearance, including hypoplastic clavicles, delayed closure of the anterior fontanelle, classic craniofacial features^22^, and dental abnormalities, such as supernumerary teeth, retained deciduous teeth, as well as impaction of multiple permanent teeth when he presented for treatment aged 10 years old^23^ (Fig. 1a,b). DFs were collected during surgical exposure of the unerupted maxillary central incisors (Fig. 1c). The patient was known to share a familial p.Ser172fs heterozygous mutation in *RUNX2* with his father, who exhibited a classic CCD phenotype^22^. Although the father had several teeth extractions, the panoramic radiograph showed at least one supernumerary tooth and one impacted permanent tooth in his mandible. (Fig. 1d).

### Biological Characterization of hDFCs from CCD Patient and Unaffected Control Individuals

**Figure 2:**
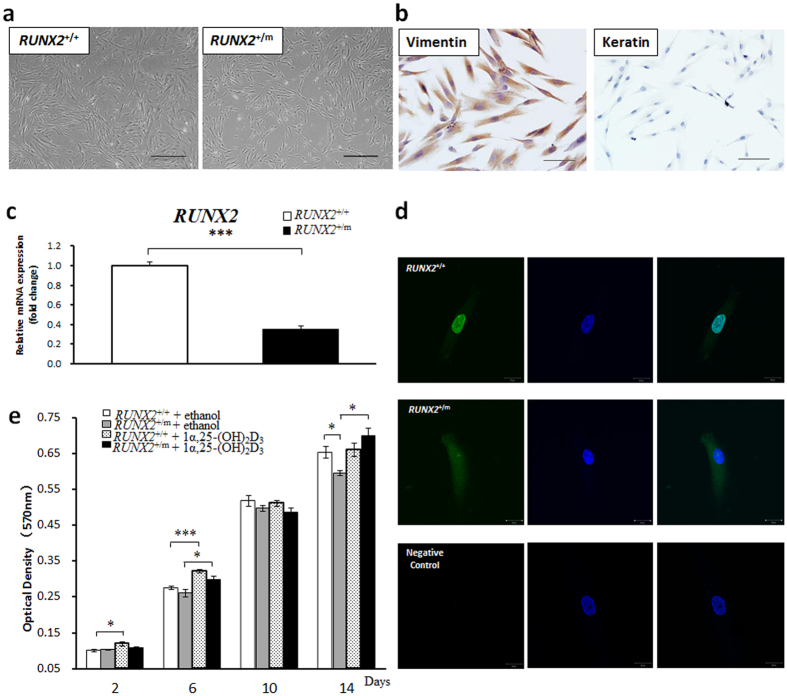
Biological characterizations of human dental follicle cells (hDFCs) from cleidocranial dysplasia (CCD) patient and control Individuals. (**a**) Representative photomicrographs of hDFCs from normal control and CCD patient, *RUNX2*^+/+^, control individuals; *RUNX2*^+/m^, CCD patient (n = 6). Scale bar = 500 μm. (**b**) Immunohistochemical staining of vimentin and keratin for CCD hDFCs (n = 3). Scale bar = 100 μm. (**c**) The *RUNX2* mRNA levels of control and CCD hDFCs were detected by Quantitative real-time polymerase chain reaction (qRT-PCR) after cultured for 3 d (n = 3). (**d**) Immunofluorescence assay of *RUNX2* for control hDFCs (upper panel) and CCD hDFCs (middle panel) (n = 3). Negative controls (lower panel) were incubated with goat IgG instead of anti-*RUNX2* as first antibody. Scale bar = 20 μm. (**e)** MTT proliferation assay for control hDFCs and CCD hDFCs at different time points treated with 10^−7^ M 1α,25-(OH)_2_D_3_ or equal volume of ethanol (n = 4). *P < 0.05. **P < 0.01. ***P < 0.001.

hDFCs isolated from the CCD patient and unaffected control individuals were plastic adherent and spindle-shaped cells, characterized by a typical fibroblast-like morphology (Fig. 2a) as described previously by Morsczeck^20^. Immunohistochemical staining showed that the CCD hDFCs were positive for vimentin but negative for keratin expression (Fig. 2b), a pattern of expression characteristic of mesenchymal cells, so as hDFCs from control individuals (data not shown). The *RUNX2* gene mutation was associated with significantly reduced *RUNX2* mRNA levels to 40% in hDFCs from the CCD patient compared to those in the unaffected controls (Fig. 2c). As a transcription factor, *RUNX2* is located mainly in the nucleus where it exerts its effects on target genes. The frameshift mutation in *RUNX2* found in our patient resulted in a truncated protein lacking nuclear-localization signal (NLS)^22^. Therefore, in contrast to the wild-type protein located in cytoplasm as well as nuclei, the mutated *RUNX2* protein expressed by the CCD hDFCs was mainly in the cytoplasm (Fig. 2d). This observation confirmed the difference in *RUNX2* expression in CCD hDFCs compared with those from the unaffected controls.

MTT cell proliferation assays showed that the proliferative capacity of CCD hDFCs was notably lower than that of the unaffected control cells at the later stage (day 14). Following 1α,25-(OH)_2_D_3_ treatment, only a slight increase in control cell number was observed, while the number of CCD hDFCs was significantly increased, eventually reaching a level equivalent to that of the control cells. Nevertheless, in other time points (day 2, day 6), 1α,25-(OH)_2_D_3_ administration enhanced control cell number with significance. (Fig. 2e).

### Expression of Osteoclast-related Genes was Restrained by *RUNX2* Mutation in Co-culture and Regulated by 1α,25-(OH)_2_D_3_ Stimulation

**Figure 3:**
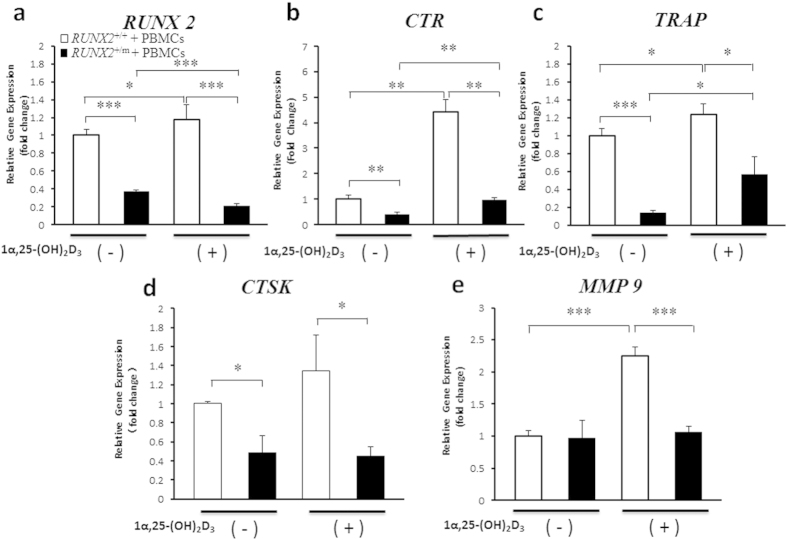
Expressions of osteoclast-related genes were restrained by *RUNX2* mutation in hDFCs and PBMCs co-culture and regulated by 1α,25-(OH)_2_D_3_ stimulation. hDFCs from normal control (*RUNX2*^+/+^) and CCD patient (*RUNX2*^+/m^) were co-cultured with peripheral blood mononuclear cells (PBMCs) in culture medium containing 1 × 10^−7^ M 1α,25-(OH)_2_D_3_ or equal volume of ethanol for 14 d (n = 4). (**a**–**e**) qRT-PCR was used to investigate the expression of *RUNX2* and osteoclast-associated genes including *CTR*, *TRAP, CTSK* and *MMP9* (n = 4). *P < 0.05. **P < 0.01. ***P < 0.001.

Fujikawa *et al*. first demonstrated that osteoblasts or mesenchyme-derived cells can induce human PBMCs to differentiate into osteoclasts^24^. We established a co-culture system of PBMCs and hDFCs to investigate the effect of the *RUNX2* gene mutation on osteoclast-related gene expression. *RUNX2* mRNA levels were decreased by 63% in CCD hDFCs compared to those in unaffected control cells (Fig. 3a). Furthermore, mRNA levels of *CTR*, *TRAP* and *CTSK* were reduced to 38%, 14% and 49% in CCD hDFCs compared with the levels in control hDFCs (Fig. 3b–d), showing a pattern of expression similar to that of *RUNX2* (Fig. 3a). In contrast, *MMP9* mRNA levels were not affected by the *RUNX2* mutation (Fig. 3e).

We further compared the two groups of cells in terms of the potential of these genes to be stimulated by 1α,25-(OH)_2_D_3_, which is a known inducer of osteoclastogenesis^18^. Showing a strong resemblance to the pattern of *RUNX2* mRNA expression (Fig. 3a), the mRNA levels of *CTR*, *CTSK*, *TRAP* and *MMP9* were upregulated (124% to 443%) in control hDFCs by 1α,25-(OH)_2_D_3_ stimulation. In contrast, following treatment of CCD hDFCs with 1α,25-(OH)_2_D_3_, only the expression of *CTR* and *TRAP* mRNA was significantly enhanced, although the levels did not reach those observed in the control hDFCs, while the expression of *CTSK* and *MMP9* was not altered (Fig. 3b–e).

### *RUNX2* Mutation Influences the Modulatory Effect of 1α,25-(OH)_2_D_3_ on the Osteoclast-inductive Capacities of hDFCs via RANKL/OPG Signaling

**Figure 4:**
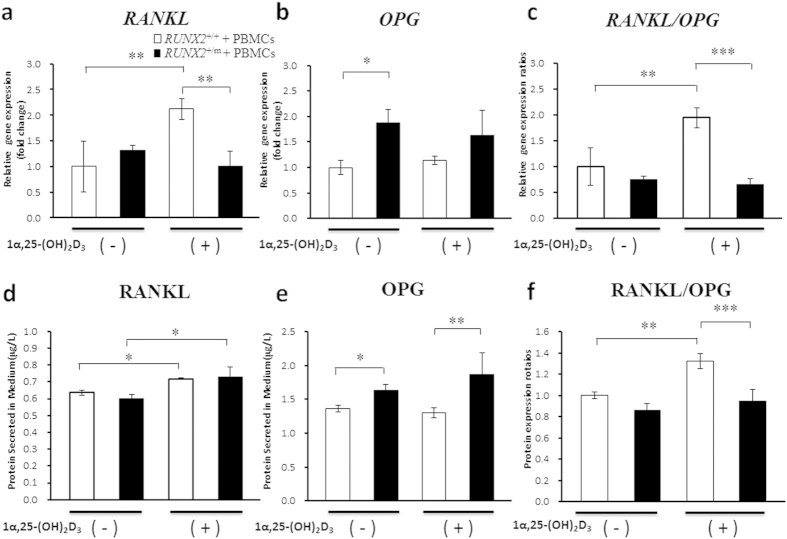
*RUNX2* mutation affects the modulation effect of 1α,25-(OH)_2_D_3_ on osteoclast-inductive capacities of hDFCs through RANKL/OPG signaling. hDFCs from normal control (*RUNX2*^+/+^) and CCD patient (*RUNX2*^+/m^) were co-cultured with PBMCs in culture medium containing 1 × 10^−7^ M 1α,25-(OH)_2_D_3_ or equal volume of ethanol for 3 or 14 d (n = 4). (**a**,**b**) qRT-PCR was used to investigate the expression of *RANKL* and *OPG* after co-culturing for 14 d (n = 4). (**c**) The ratio of RANKL/OPG mRNA expression in hDFCs on 14 d (n = 4). (**d**,**e**) ELISA assay was used to analyze RANKL and OPG protein concentration in conditioned medium after co-culturing for 3 d (n = 4). (**f**) The ratio of RANKL/OPG protein concentration in conditioned medium on 14 d (n = 4). *P < 0.05. **P < 0.01. ***P < 0.001.

RANKL and its inhibitor OPG play important roles in bone metabolism. In our study, both groups of hDFCs expressed comparative *RANKL* mRNA levels (Fig. 4a), while CCD hDFCs expressed 88% higher levels of *OPG* mRNA than those from the unaffected controls (Fig. 4b). The basal RANKL and OPG protein levels measured in the conditioned media of cultured CCD hDFCs and control cells were consistent with the mRNA findings. When stimulated with 1α,25-(OH)_2_D_3_, the *RANKL* mRNA level increased to 210% in control hDFCs but decreased by 24% in CCD hDFCs, while the protein concentrations in conditioned media obtained from the two groups of hDFCs showed closely analogous (Fig. 4a,d). Notably, *OPG* expression in both groups was unaffected by 1α,25-(OH)_2_D_3_ stimulation at both the mRNA and protein levels (Fig. 4b,e). Accordingly, the RANKL/OPG ratios were slight and significant lower in CCD hDFCs comparing with normal cells at the level of mRNA and protein, respectively (Fig. 4c,f). In contrast, 1α,25-(OH)_2_D_3_ stimulation significantly altered the RANKL/OPG ratios at the mRNA and protein levels in normal hDFCs, but had no effect on the corresponding ratios in CCD hDFCs (Fig. 4c,f). All these results indicated that the impairment of the osteoclastogenic capacity of CCD hDFCs with a p.Ser172fs mutation in *RUNX2*, is mediated at least partially by disturbances in the RANKL/OPG signaling pathway and cannot be completely rescued by 1α,25-(OH)_2_D_3_ stimulation.

### 1α,25-(OH)_2_D_3_ Strongly Facilitated Osteoclast Formation Induced by Unaffected control hDFCs but not by CCD hDFCs

**Figure 5:**
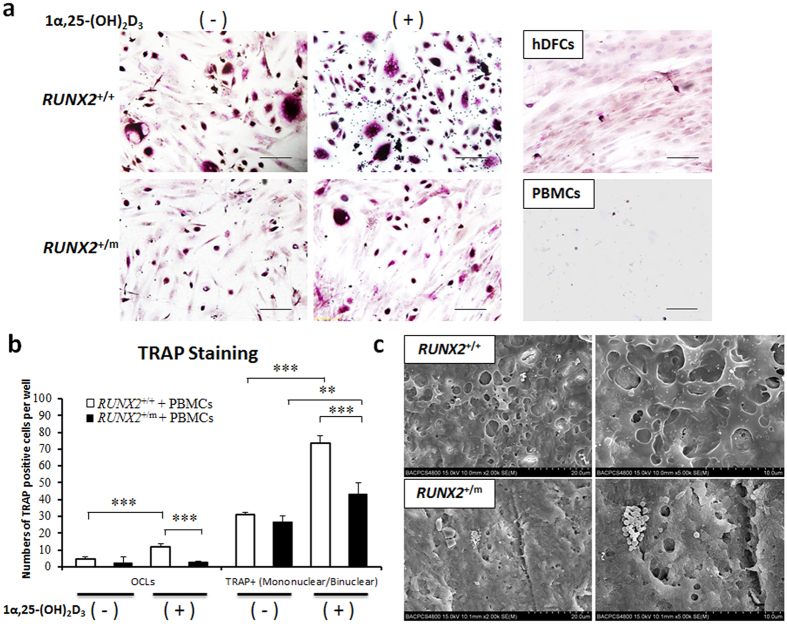
1α,25-(OH)_2_D_3_ strongly facilitated osteoclast formation induced by *RUNX2*^+/+^ hDFCs but not by *RUNX2*^+/m^ hDFCs. hDFCs from normal control (*RUNX2*^+/+^) and CCD patient (*RUNX2*^+/m^) were co-cultured with PBMCs in culture medium containing 1 × 10^−7^ M 1α,25-(OH)_2_D_3_ or equal volume of ethanol for 16 d or 21 d. (**a**) TRAP straining of the co-cultures of PBMCs and *RUNX2*^+/+^ or *RUNX2*^+/m^ hDFCs in the stimulation of 1α, 25-(OH)_2_D_3_ on 16 d (n = 4). Scale bar = 100 μm. (**b**) The quantification of TRAP straining for PBMCs and hDFCs co-cultures. Eight sites on each coverslip were measured for the number of TRAP^+^ multinucleated cells (≥3 nuclei per cell) and TRAP^+^mononuclear / binuclear cells on 16 d (n = 4). (**c**) Scanning electron microscopy (SEM) analyses for hDFCs and PBMCs co-cultures. hDFCs and PBMCs were co-cultured on bovine cortical bone slices (6 × 6 mm^2^) for 21 d with 1 × 10^−7^ M 1α,25-(OH)_2_D_3_ . SEM was used to visualize resorption lacunae on bone slices (n = 3). **P < 0.01. ***P < 0.001.

Considering the limited OCLs observed when hDFCs were co-cultured with PBMCs in the absence of cytokines, the number of TRAP^+^ mononuclear/binuclear cells was also quantified to evaluate the ability of unaffected control and CCD hDFCs to induce osteoclast formation (Fig. 5a). At the basal level, the control hDFCs exhibited a slightly higher number of TRAP^+^ cells comparing with the CCD hDFCs (Fig. 5a,b). In parallel with the expression of genes beneficial for osteoclastogenesis, the formation of OCLs and TRAP^+^ mononuclear/binuclear cells was highly favored in normal hDFCs stimulated with 1α,25-(OH)_2_D_3_. In contrast, only the number of TRAP^+^ mononuclear/binuclear cells was significantly increased in CCD hDFCs (Fig. 5b).

Observation of bone absorption with SEM was performed to investigate the functionality of osteoclasts produced in the co-culture systems. Bone lacunae were observed on bone slices cultured with hDFCs and PBMCs in the presence of 1α,25-(OH)_2_D_3_ (Fig. 5c), while almost none were observed in the absence of 1α,25-(OH)_2_D_3_ (data not shown).

## Discussion

In our previous study, we identified a heterozygous frameshift mutation in *RUNX2* gene of the CCD patient in this study and showed that absence of the NLS resulted in inadequate nuclear accumulation^22^. In the present study, we showed a 60% reduction in *RUNX2* mRNA expression with a concomitant reduction in the nuclear distribution of the *RUNX2* protein in CCD hDFCs. Furthermore, our results indicated that the diminished proliferative and osteoclastogenic capacity of hDFCs caused by the truncated *RUNX2* protein contributes to the delay in permanent teeth eruption in CCD patients. Although the proliferation of CCD hDFCs was promoted to a comparable level as control hDFCs under 1α,25-(OH)_2_D_3_ administration, its osteoclastogenic ability cannot be rescued.

Tooth eruption is a time-dependent and localized event requiring not only alveolar bone resorption, but also bone formation regulated by spatiotemporal-specific expression of *RUNX2* and other associated genes in DFs^12^,^25^. DFs are critically involved in this process^11^, through keeping an accurate balance between osteoblasts and osteoclasts. RANKL and CSF-1, secreted primarily by DFCs or other stromal/osteoblastic cells during tooth eruption, had been demonstrated to be two main molecules promoting mononuclear cell recruitment and fusion into functional osteoclasts^13^,^26^. The *in vitro* osteoclast formation system of co-culture bone marrow stromal cells/osteoblasts with osteoclast precursors (bone marrow cells, PBMCs) without additional cytokines was well established^26^,^27^,^28^. Various types of cells derived from human dental tissue, such as dental pulp cells (DPCs)^23^, periodontal ligament cells (PDLCs)^29^,^30^ and DFCs^29^,^30^ were also confirmed to have osteoclast-inductive capacity under similar condition recently. In this study, using a hDFCs and PBMCs co-culture system, we found significantly reduced expression of the osteoclast-associated markers *TRAP*, *CTR* and *CTSK* in CCD hDFCs, with a corresponding decrease in the number of OCLs. These observations indicate the impaired osteoclastogenesis-inductive ability of hDFCs in association with *RUNX2* mutation and is consistent with a previous report that heterozygous *RUNX2* mutation in mice is associated with insufficient osteoclast recruitment in tooth eruption pathway^10^. In accordance with our results, another study also showed weaker matrix remolding and osteoclast-inductive capacities in CCD hDFCs when co-cultured with mouse bone marrow cells^31^. Studies have also demonstrated that *Runx2* mutation significantly impairs the capacity for induction of osteoclast differentiation in human DPCs, human PDLCs, as well as mouse calvaria cells^31^,^32^.

RANKL/RANK/OPG signaling is considered as the typical pathway modulating bone remodeling. OPG, a soluble decoy receptor for RANKL, which is also secreted by osteoblasts, prevents the osteoclastogenic effect. Consequently, the RANKL/OPG ratio plays a crucial role in adjusting the localized balance between alveolar bone formation and resorption^14^. Although the precise role of *RUNX2* in osteoclast differentiation is controversial, its effect on RANKL and OPG regulation has been demonstrated. Previous studies have shown that *RUNX2*-binding elements are present in the rat, mouse and human *RANKL* genes, as well as in the human *OPG* promoter^33^,^34^. A *RUNX2*^−/−^ calvaria-derived cell line co-cultured with normal bone marrow cells expressed *OPG* strongly, while *RANKL* levels were negligible. Adenoviral introduction of *RUNX2* into this cell line resulted in upregulated *RANKL* expression and downregulated *OPG* expression, which restored osteoclast differentiation^35^. These findings indicated that *RUNX2* promotes osteoclast differentiation by increasing the RANKL/OPG ratio. In this study, we found that, although RANKL expression was not affected by *RUNX2* mutation in hDFCs, OPG levels were upregulated. Accordingly, the RANKL/OPG ratio was significantly decreased by *RUNX2* mutation. In accordance with our data, other studies have demonstrated reduction in the RANKL/OPG ratio as well as the osteoclast-inducing ability of *RUNX2* mutant hDFCs, hPLCs and hDPCs^15^,^23^,^31^.

1α,25-(OH)_2_D_3_ is a steroid hormone derivative that plays a pivotal role in bone metabolism. Although the precise mechanism is not clear, evidence reveals that 1α,25-(OH)_2_D_3_ has direct effects on RANKL-induced osteoclast formation in multiple cell types, but not in hDFCs^36^,^37^,^38^. In the present study, we demonstrated that the increases in *RANKL* mRNA levels and the *RANKL*/*OPG* ratio observed following 1α,25-(OH)_2_D_3_ treatment were reflected in the increased numbers of OCLs in co-cultures of control hDFCs and PBMCs. In contrast, *RANKL* levels and the number of OCLs in co-cultures of CCD hDFCs and PBMCs were not affected by 1α,25-(OH)_2_D_3_ treatment, indicating that *RUNX2* haploinsufficiency abolishes 1α,25-(OH)_2_D_3_-induced *RANKL* gene activation in hDFCs. Although a response element for 1α,25-(OH)_2_D_3_ has been identified in the *RUNX2* promoter^39^, the role of *RUNX2* in 1α,25-(OH)_2_D_3_-induced RANKL expression remains controversial. Some previous studies indicated that *RUNX2* is not essential for 1α,25-(OH)_2_D_3_-induced RANKL expression in osteoblastic cells, since *runx2*-deficent calvarial cells induced the formation of osteoclasts in co-culture with mouse spleen cells due to stimulation of RANKL expression^40^. In contrast, *RUNX2* knockdown and overexpression studies revealed that RANKL expression in stromal/osteoblastic cells is regulated by 1α,25-(OH)_2_D_3,_ which transactivates the gene predominantly via the *RUNX2*-dependent pathway, but also via a *RUNX2*-independent mechanism that depends on direct binding of the VDR complex to the VDR element in the *RANKL* promoter^40^. Thus, 1α,25-(OH)_2_D_3_ enhances the osteoclast-inductive capacity by increasing the RANKL/OPG ratio in normal hDFCs and this effect is eliminated by *RUNX2* mutation in CCD hDFCs.

In conclusion, in this study, we show that hDFCs derived from CCD patient, a natural model for studying *RUNX2* and obstacles to dental eruption, exhibit reduced proliferative capacity compared with unaffected hDFCs. In addition, *RUNX2* mutation causes impaired dental follicle-induced osteoclastogenesis *in vitro* by increasing OPG expression and decreasing the RANKL/OPG ratio, which is a potential mechanism underlying delayed permanent teeth eruption in CCD patients. Furthermore, this deficiency is not rescued by 1α,25-(OH)_2_D_3_ application because 1α,25-(OH)_2_D_3_-induced RANKL activation in hDFCs is mediated by *RUNX2*.

## Methods

### Primary hDFCs Isolation and Culture

A 10-year old Chinese boy who was clinically and genetically diagnosed with CCD^22^ and three unaffected children (aged 8–12 years) who attended the clinic for orthodontic reasons were examined and treated with informed parental consent. This project was approved by the Ethical Committee of Peking University School of Stomatology (approval No. PKUSSIRB-2012004). All experiments in this study were carried out in accordance with the approved guidelines.

Briefly, the DFs were isolated during surgical exposure of the impacted maxillary central incisors. Soon after harvesting, DFs were digested in a mixture of 3 mg/mL collagenase type I (Sigma-Aldrich, MO, USA) and 4 mg/mL dispase (Sigma-Aldrich) for 1 h at 37 °C to generate single cell suspension. The cells were then cultured in DMEM medium (Gibco, MO, USA) containing 10% fetal bovine serum (FBS; Hyclone, UT, USA) at 37 °C in a humidified atmosphere containing 5% CO_2_. The medium was changed twice each week and hDFCs between passages 3 and 6 were used in experiments.

### PBMCs Isolation

Whole human peripheral blood obtained from two healthy volunteers was layered on Ficoll-Paque PLUS density gradient media (GE Healthcare, USA) and separated by centrifugation at 400 × *g* for 30 min. The peripheral blood monocyte layer was collected and finally recovered in culture medium as PBMCs.

### Co-culture of hDFCs and PBMCs

hDFCs were seeded onto coverslips or bovine cortical bone slices in 24-well plates (3.5 × 10^4^ cells/well) for 1 day. Then PBMCs at a density of 2.5 × 10^6^ cells/well were layered on top of the hDFCs and cultured in DMEM (containing 10% FBS) with 1 × 10^−7^ M 1α,25-(OH)_2_D_3_ (Sigma-Aldrich) or an equal volume of vehicle (ethanol) for the indicated time-points.

### Immunohistochemical Staining and Immunofluorescence Assay

hDFCs were seeded onto coverslips in 24-well plates at a density of 2 × 10^4^ cells/well and cultured to 80% confluence. The cells were then fixed in 4% paraformaldehyde (Sigma-Aldrich) for 40 min, and permeabilized with 0.2% Triton X-100 (Sigma-Aldrich) in PBS for 15 min. Next, the cells were blocked, and then incubated with primary antibodies for anti-keratin, anti-vimentin (Zhongshan Bioengineering Co. Ltd., China) and anti-*RUNX2* (Santa Cruz Biotechnology, CA, USA) overnight at 4 °C. The rest of the procedure of immunohistochemical staining for keratin and vimentin was performed using an SP immunohistochemical kit and a 3,3′-diaminobenzidine coloration kit (Zhongshan Bioengineering Co. Ltd.) according to the manufacturer’s instructions. Squamous carcinoma cells and adipocytes were used as positive controls for keratin and vimentin staining, respectively. For immunofluorescence analysis of *RUNX2*, green-fluorescent fluorescein isothiocyanate (FITC)-conjugated rabbit anti-goat antibody (Zhongshan Bioengineering Co. Ltd.) was used at a dilution of 1:100. Coverslips were mounted with DAPI-containing mounting media, and examined with a Zeiss LSM 5 EXCITER confocal microscopy (Carl Zeiss, Germany). The cells incubated with goat IgG instead of anti-*RUNX2* as first antibody were used as negative controls.

### MTT Assay for Cell Proliferation

hDFCs were plated in 96-well plates at a density of 5 × 10^3^ cells/well with 1 × 10^−7^ M 1α,25-(OH)_2_D_3_ or ethanol. At the indicated time-points, 200 μl fresh medium was replaced before 20 μl of MTT solution (5 mg/ml; Sigma) was added to each well. After incubation for 4 h, 150 μl dimethyl sulfoxide (Sigma-Aldrich) was added and mixed by repeated pipetting to dissolve the formazan-salt. Absorbance was measured at 490 nm, while the background absorbance was measured at 630 nm using an ELx808 absorbance microplate reader (BioTeK, USA).

### Quantitative Real-time Polymerase Chain Reaction (qRT-PCR)

Total RNA was isolated with TRIzol reagent (Invitrogen, CA, USA) from cultured hDFCs or from co-cultured cells after 3 d or 14 d. TaqMan Reverse Transcription Reagents (Applied Biosystems, CA, USA) were used to reverse-transcribe mRNA into cDNA. qRT-PCR was then performed with the Applied Biosystems 7500 Real-Time PCR System (Applied Biosystems, CA, USA) using a SYBR Green PCR kit (Roche Applied Science, IN, USA) according to the protocol, to investigate the expression of osteoclast-associated genes (*CTR*, *CTSK*, *MMP9* and *TRAP*), *RANKL*, *OPG*, and *RUNX2* (Table 1).

### TRAP Staining and Quantification

After co-culture of hDFCs and PBMCs for 16 days, TRAP staining was performed with Acid Phosphatase, Leukocyte (TRAP) Kit (387A; Sigma-Aldrich, USA) according to the manufacturer’s instructions. Nuclei were stained with hematoxylin. The TRAP^+^ multinucleated cells (≥3 nuclei per cell; osteoclast-like cells, OCLs) and TRAP^+^ mononuclear/binuclear cells were enumerated in eight sites (magnification, ×200) on each coverslip.

### ELISA

hDFCs were co-cultured with PBMCs for 3 days. The levels of RANKL and OPG in the conditioned medium were analyzed with commercial ELISA kits (DY626, DY805; R&D System, USA) according to the manufacturers’ protocols. The optical density (OD) value at 450 nm was determined using an ELx808 absorbance microplate reader (BioTeK).

### Bone Resorption and Scanning Electron Microscopy (SEM)

hDFCs and PBMCs were grown on bovine cortical bone slices (6 × 6 mm^2^) for 21 days. To visualize resorption lacunae by SEM, bone slices were fixed in 2.5% glutaraldehyde for 7 min before sonication in 0.25 mol/L ammonium hydroxide and distilled water successively. This was followed by graded ethanol series dehydration. Slices were then coated with gold and examined with a S4800 FESEM (Hitachi, Japan) operating at 15 kV.

### Statistical Analysis

Statistical analyses were carried out with SPSS 19.0. Difference between two groups was performed using two-tailed Student’s *t* test. Comparisons among multiple time points or treatments were determined by one-way analysis of variance (ANOVA), followed by a multiple-comparison with the Bonferroni’s post-hoc test or the Games-Howell post-hoc test. Data represent the average of a minimum of three independent experiments expressed as means ± standard deviation (s.d.). The level of statistical significance was considered as *P* < 0.05.

## Additional Information

**How to cite this article**: Wang, X. Z. *et al*. *RUNX2* Mutation Impairs 1α,25-Dihydroxyvitamin D_3_ mediated Osteoclastogenesis in Dental Follicle Cells. *Sci. Rep*. **6**, 24225; doi: 10.1038/srep24225 (2016).

## Figures and Tables

**Table 1:** Primer sequences of *CTR, CTSK, MMP9, TRAP, RANKL, OPG*, and *RUNX2* used in Quantitative real-time polymerase chain reaction (qRT-PCR).

Gene	Sense	Antisense
*GAPDH*	5′-CGACAGTCAGCCGCATCTT-3′	5′-CCAATACGACCAAATCCGTTG-3′
*CTR*	5′-TGGTGCCAACCACTATCCATGC-3′	5′-CACAAGTGCCGCCATGACAG-3′
*CTSK*	5′-TGAGGCTTCTCTTGGTGTCCATAC-3′	5′-AAAGGGTGTCATTACTGCGGG-3′
*MMP9*	5′-GTGCTGGGCTGCTGCTTTGCTG-3′	5′-GTCGCCCTCAAAGGTTTGGAAT-3′
*TRAP*	5′-GACCACCTTGGCAATGTCTCTG-3′	5′-TGGCTGAGGAAGTCATCTGAGTTG-3′
*RUNX2*	5′-GCGTCAACACCATCATTCTG-3′	5′-CAGACCAGCAGCACTCCATC-3′
*RANKL*	5′-CACTATTAATGCCACCGAC-3′	5′-GGGTATGAGAACTTGGGATT-3′
*OPG*	5′-AGAGAAAGCGATGGTGGATG-3′	5′ –CGGTGGCATTAATAGTGAGATG-3′
